# Effect of School HPV Vaccination Requirements on Pediatricians’ Recommendations †

**DOI:** 10.3390/vaccines12121374

**Published:** 2024-12-06

**Authors:** Ashley Hedrick McKenzie, Lara S. Savas, Ross Shegog, Dale S. Mantey, Erica L. Frost, Paul Gerardo Yeh, C. Mary Healy, Stanley Spinner, L. Aubree Shay, Sharice M. Preston, Sally W. Vernon

**Affiliations:** 1 Department of Communication, Clemson University, 105 Sikes Hall, Clemson, SC 29634, USA; 2 School of Public Health, University of Texas Health Science Center at Houston, 7000 Fannin St., Houston, TX 77030, USA; 3 The Michael & Susan Dell Foundation for Healthy Living, School of Public Health, University of Texas Health Science Center at Houston, 700 Fannin St., Houston, TX 77030, USA; 4 Department of Pediatrics, Infectious Diseases Section, Baylor College of Medicine, 1 Baylor Plaza, Houston, TX 77030, USA; 5 Texas Children’s Pediatrics, 1919 S Braeswood Blvd, Houston, TX 77030, USA

**Keywords:** HPV vaccines, human papillomavirus (HPV), vaccination, health care provider communication, physician barriers, intervention strategies

## Abstract

**Background/objectives**: Pediatrician recommendations are highly influential in parents’ decisions to vaccinate their children against HPV. Unqualified, presumptive, and bundled recommendations (UPBRs) are associated with increased HPV vaccine uptake and are considered best practice. This study analyzes pediatricians’ self-reported data to assess changes in UPBR use and the psychosocial determinants of UPBR use as a result of the implementation of a multi-level intervention, the Adolescent Vaccination Program (AVP). **Methods**: We surveyed pediatricians across 51 clinics in the Houston area. Baseline surveys (*n* = 137) were distributed in 2015, and follow-ups (*n* = 120) in 2019. **Results**: Pediatrician UPBR use significantly increased as a result of AVP implementation. Change in the provider belief that it is necessary to tell parents that HPV vaccination is not required for public school attendance significantly predicted UPBR use at follow up. This belief was also a significant mediator of increased use of UPBRs at follow-up. **Conclusions**: AVP was successful in increasing pediatricians’ use of UPBRs. Change in UPBR use is related to one critical psychosocial determinant: beliefs about communication regarding the non-mandatory nature of HPV vaccination for school enrollment. HPV vaccine promotion efforts should devote focus to changing pediatricians’ beliefs about the necessity of disclosing the non-mandatory nature of HPV vaccination for school attendance.

## 1. Introduction

An estimated 42.5 million people in the United States (U.S.) were infected with human papillomavirus (HPV) in 2018, making HPV the most common sexually transmitted infection in the U.S. [1]. HPV is a leading cause of cervical, anal, vaginal, oropharyngeal, vulvar, and penile cancers [2]. HPV vaccination effectively prevents high-risk strains of HPV that can cause cancer if infection persists. HPV vaccination is recommended for children ages 11–12 [3]. However, only 58.6% of adolescents aged 13 through 15 have completed the HPV vaccine series [4], in contrast with the U.S. Department of Health and Human Services’ Healthy People 2030 goal of 80% completion [5]. Rates of initiating the HPV vaccine series, or receiving at least one dose, also remain low; 78.5% of U.S. girls and 75.4% of boys between 13–17 years old have initiated the HPV vaccine series [6].

Pediatricians’ recommendations are highly influential in parents’ decisions to have their children receive the HPV vaccine [7,8,9,10]. Unsurprisingly, the quality of pediatricians’ recommendations also impacts parents’ vaccine decision making [8,11,12]. Current CDC guidelines identify a “presumptive, bundled” recommendation as best practice [13]. A clinical trial found that use of presumptive, bundled recommendations is associated with increased HPV vaccine coverage [14]. Presumptive recommendations use language that reflects the assumption that the child will receive the vaccine (e.g., “Your child is due for the HPV vaccine today”). Ample research has evaluated the efficacy of presumptive HPV vaccine recommendations in US clinical settings [14,15,16,17,18]. Bundling refers to the practice of recommending the HPV vaccine alongside other routine vaccines for 11–12-year-olds, specifically between Tdap and meningococcal vaccinations [13]. Bundling is also associated with improved HPV vaccine outcomes [19,20,21,22]. In addition to presumptive language and bundling, providing unqualified recommendations—which do not present the HPV vaccine as optional or different from other vaccines—is another best practice for physicians to use when discussing the HPV vaccine with parents [11]. Types of provider qualifications that weaken HPV vaccine recommendations include emphasizing parental choice, advising that the HPV vaccine is not required for school attendance, and explaining that vaccination can be delayed [12]. Unqualified, presumptive, and bundled recommendations (UPBRs) are associated with a greater likelihood of adolescent vaccine initiation [11,23].

However, pediatricians may experience a variety of barriers to delivering high-quality recommendations. Physicians’ self-reported barriers to giving vaccine recommendations, such as concerns about vaccine safety and efficacy, concerns about the financial burden of the vaccine on parents, and concerns about parents’ negative attitudes about the vaccine, are associated with lower rates of delivering HPV vaccine recommendations and HPV vaccine initiation among adolescent patients [24,25,26].

Given the importance of pediatrician recommendations and the barriers to delivering effective recommendations that they may experience, the Adolescent Vaccination Program (AVP)—a multi-component and multi-level intervention developed to increase HPV vaccination among adolescents—includes a suite of evidence-based strategies, including education for pediatricians that is certified for a continuing medical education credit (CME), aimed at improving the quality and consistency of pediatricians’ HPV vaccination recommendations [27,28]. AVP’s multi-component approach to delivering evidence-based strategies aimed at clinic systems and physicians is supported by research findings that, while CME alone is not sufficient to change physicians’ attitudes, CME is effective when combined with system-level strategies such as assessment and feedback, provider reminders, and/or patient reminders and recall [29,30,31]. AVP’s evidenced-based strategies include: HPV immunization champions, designated clinic staff members who facilitate intervention implementation; CME for pediatricians and nurses about HPV vaccination, including didactic information about using UPBRs and a simulated clinic room interaction quiz; pediatrician assessment and feedback; prompts to remind providers about patient (child) vaccine eligibility; tailored patient (parent) reminders and recall; and strong leadership support, including network-wide messages from the medical director in support of AVP and use of UPBRs [27,28]. These strategies were designed using Intervention Mapping [32], a systematic intervention planning approach informed by evidence and behavioral theory, to target clinic system processes and pediatricians’ psychosocial barriers to delivering vaccination recommendations, including a range of normative beliefs, areas of knowledge, efficacy-based barriers, and time-related barriers [27]. System-level AVP strategies were designed to help providers overcome barriers and integrate strong, consistent vaccine practices into routine care. AVP also addressed providers’ concerns about communicating with parents about the HPV vaccine and reinforced their ethical responsibility to recommend the vaccine based on patient benefit and public health guidelines [27,33], regardless of school requirements, through consistent messaging in CME training, simulated interactions, and leadership communications.

Three-year evaluation study results showed that AVP intervention effectively increased HPV vaccination rates in a 51-clinic network by 37% [28]. Examination of provider recommendation style in the AVP study also provided evidence that pediatricians’ use of UPBRs was associated with significantly higher HPV vaccine uptake [11]. However, AVP’s impact on pediatrician attitudes and beliefs that may influence UPBR adoption has not yet been investigated. While existing research has explored a range of psychosocial variables impacting pediatricians’ willingness to recommend the HPV vaccine [25,26,34,35], less is known about what pediatrician attitudes and beliefs may influence UPBR use. 

Therefore, the current study uses data from the 3-year evaluation of AVP [28] to investigate AVP’s effect on UPBR use, AVP’s effect on potential psychosocial determinants of UPBR use, and potential mechanisms related to pediatrician recommendation style. Specifically, this study describes (1) changes in UPBR use as a result of AVP implementation, (2) changes in psychosocial determinants of pediatrician UPBRs, (3) determinants which are most predictive of UPBRs, and (4) determinants that have mediated change toward UPBRs as a result of AVP implementation. Analyses are not stratified by AVP’s components (e.g., CME, provider assessment and feedback, provider reminders, patient reminders, and parent education); the results report the collective impact of all AVP’s bundled strategies. By determining the psychosocial mechanisms of change between the AVP program and pediatrician UPBR use, these results provide evidence of effective strategies to target provider recommendation behaviors and can guide the design of future interventions to improve providers’ vaccine recommendations. In this study, the results presented in [36] are expanded upon.

## 2. Methods

### 2.1. Overview

We surveyed and delivered the AVP to pediatricians of patients between the ages of 11 and 17. Pediatricians completed baseline (*n* = 137) and follow-up surveys (*n* = 120) before and after the delivery of the AVP, an intervention aimed at increasing the uptake of the HPV vaccine. For the mediation analysis, we also conducted an additional subsample analysis on *n* = 66 pediatricians who completed both baseline and follow-up surveys. Therefore, this subsample of pediatricians experienced the entirety of AVP implementation between 2015 and 2019.

The data were collected as part of a larger study conducted within 51 clinics comprising a large pediatric network. This network has over 200 board-certified pediatricians and serves a diverse population in the greater Houston, Texas, USA area. This project was approved by the University of Texas Health Science Center Institutional Review board (HSC-SPH—14-0725).

### 2.2. Data Collection

All network-affiliated pediatricians received an email link to complete the baseline survey online between August and September 2015. At this time, 249 pediatricians were affiliated with the network. Pediatricians were offered a USD 50 gift card as an incentive to complete the survey, which took under 30 min to complete. The same recruitment procedure was followed for the post-survey between January and February 2019.

### 2.3. Measures

#### 2.3.1. Pediatrician Demographic Variables

Demographic measures included: race/ethnicity, sex, age, number of patients seen on a typical day (less than 15; 15–19; 20–29; 30 or more), number of years worked at the clinic, and year that residence training was completed.

#### 2.3.2. Pediatrician Psychosocial Variables

In total, 34 survey items measured a range of pediatrician psychosocial constructs that may function as barriers or facilitators to delivering HPV vaccine recommendations. All survey items were analyzed individually due to low reliability among survey items in some constructs. Psychosocial constructs included pediatricians’ knowledge and attitudes, perceptions of parental attitudes, concerns about practicality, feelings about school requirements, perceptions of physician norms, communication self-efficacy, and self-efficacy to communicate about sex. Section 3.3 lists the psychosocial constructs assessed in this study and corresponding survey measures. Survey items were selected from existing valid and reliable scales from a variety of sources [34,35,37,38,39,40,41,42,43,44] on the basis of face validity and organized into constructs. Some of the proposed constructs did not have acceptable reliability among survey items (see Section 3.3 for Cronbach’s alphas for each construct). Therefore, all analyses examining pediatrician psychosocial variables were conducted at the item level. Significance criteria for these analyses were adjusted for Type 1 error using the Bonferroni correction method [45]. 

#### 2.3.3. Pediatrician Communication Strategies

To assess pediatrician communication strategies for recommending the HPV vaccine, the survey included the following question: “Choose the statement that is closest to how you typically introduce adolescent vaccinations during a pediatric patient visit”. Response choices were, (1) “Your child is due for three vaccines, including the HPV vaccine”; (2) “Your child is due for two vaccines, Tdap and meningococcal. There is also the HPV vaccine, which is optional”; (3) “Your child is due for three vaccines: Tdap, HPV, and meningococcal vaccine”; (4) “Your child is due for Tdap and meningococcal vaccine, and we can discuss the HPV vaccine if you like”; and (5) “Other”. The third response choice represents the UPBR, which is most effective in achieving adolescent HPV vaccination [11]. Selection of the third answer choice was coded as “1” to reflect pediatrician use of UPBRs. Selection of any other answer choice was coded as a “0” to represent non-use of UPBRs.

#### 2.3.4. Mediation Variables

Three variables were selected for use in the mediation analyses. The predictor variable was non-use of UPRs at baseline, and the dependent variable was use of UPBR at follow-up (see Section 2.3.3. for detail about construction of UPBR use and UPBR non-use). In other words, the mediation model sought to identify variables that explained why pediatricians who did not use UPBRs at baseline changed to using UPBRs after AVP implementation. Out of the 34 psychosocial variables, only one variable met the criteria for use as a mediator in the model (see Section 2.4 for selection criteria). This variable was pediatricians’ agreement with the statement, “I feel I have to tell parents the HPV vaccine is not required for school”. Responses ranged from 1 = “strongly disagree” and 4 = “strongly agree”. Disagreement with this statement (strongly disagree and somewhat disagree) at the follow-up survey (after AVP implementation) was entered into the model as a mediator.

### 2.4. Data Analysis

The analysis was comprised of a chi-square test to detect the change in UPBR use between baseline and follow-up surveys (Section 3.2); t-tests to determine item-level change between baseline and follow-up surveys (Section 3.3); and logistic regression to determine which psychosocial variables predicted UPBR use (Section 3.4). For these analyses, the Bonferroni correction method was applied to account for Type 1 errors. Results from each analysis were considered significant if *p* ≤ 0.05/number of comparisons [45]. Analyses included 34 different psychosocial variables, so the significance level was set at *p* ≤ 0.05/34, or *p* ≤ 0.0015.

Last, we conducted two mediation models, with UPR use at follow-up as the model outcome. Psychosocial variables were entered in the model as mediators if: (1) the variable significantly changed between baseline and follow-up and (2) the variable significantly predicted the use of UPBRs. Only one variable met these criteria: disagreement with feeling compelled to disclose school vaccine requirements to parents. The first mediation model was calculated with the full sample of all pediatricians in the network who completed a survey (*n* = 137 at baseline, before AVP implementation and *n* = 120 at follow-up, after AVP implementation). The second mediation model was calculated using only the subsample of pediatricians who completed both the baseline and follow-up surveys (*n* = 66). In other words, these pediatricians experienced the full duration of AVP implementation between 2015 and 2019. 

## 3. Results

### 3.1. Participants

The sample consisted of 137 eligible pediatricians at baseline and 120 at follow-up. Surveys were cross-sectional, meaning that baseline and follow-up samples were not matched (except for final mediation analysis, matched subsample of *n* = 66 pediatricians). Of the 189 unique pediatricians who completed at least one survey, 36% completed both the baseline and follow-up surveys. Respondents were mainly white females (Table 1). The average pediatrician age was 47, and the average year of completing residency training was 2002. 

### 3.2. Change in Use of UPBRs

At baseline, 51.85% of pediatricians reported using UPBRs (*n* = 70). At follow-up, 84.17% (*n* = 101) of pediatricians reported using UPBRs. A chi-square test of independence showed that pediatricians at follow-up were significantly more likely than pediatricians at baseline to report using UPBRs (*X*^2^ (254) = 30.03, *p* < 0.001).

### 3.3. Changes in Psychosocial Variables

AVP implementation was associated with a statistically significant change in six (17.6%) of the measured psychosocial determinants for physician use of UPBRs (Table 2). 

The following psychosocial determinants significantly decreased between baseline and follow-up, meaning they were less of a barrier to UPBR use after the implementation of AVP: pediatrician belief that it is okay to delay HPV vaccination for 11–12 year-olds until they are older (*p* < 0.001); pediatrician concern about the financial burden of the HPV vaccine on parents (*p* < 0.001); pediatrician belief that it is necessary to tell parents that the HPV vaccine is not required for school (*p* < 0.001); and pediatrician belief that it is necessary to discuss issues of sexuality when recommending the HPV vaccine to patients or parents of patients (*p* = 0.001). 

Physicians rated the following psychosocial determinants with significantly higher levels of agreement at follow-up compared to those who completed baseline surveys: the belief that the severity of HPV-associated disease in males justifies the routine use of the HPV vaccine in males (*p* < 0.001) and the rated importance of ensuring that adolescents are up to date with HPV vaccination (*p* < 0.001).

### 3.4. Variables Predicting UPBR Use 

Twenty-six predictor variables were removed due to multicollinearity, resulting in eight final predictors used in the logistic regression (Table 3). Change in one pediatrician belief significantly predicted UPBR use: it is necessary to tell parents that HPV vaccination is not required for school. Specifically, an increase in disagreement with the statement was associated with the use of UPBR.

### 3.5. Mediators Explaining AVP Efficacy

**Figure 1:**
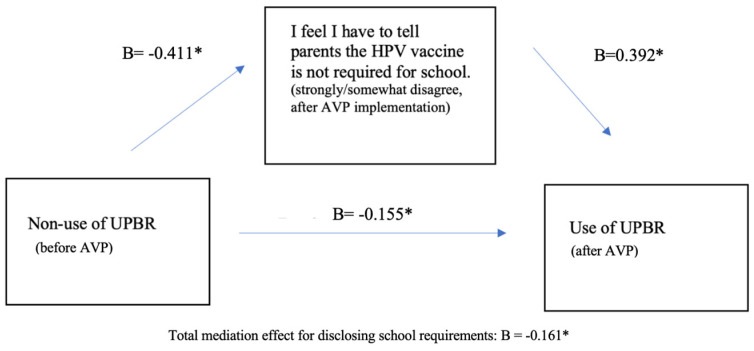
A mediation analysis of change in use of unqualified, bundled, presumptive recommendation (UPBR) before and after delivery of AVP. * denotes *p* < 0.05.

Psychosocial variables were entered in the model as mediators if: (1) the variable significantly changed between baseline and follow-up and (2) the variable significantly predicted the use of UPBRs. One psychosocial variable met these criteria: pediatricians’ belief that it is necessary to tell parents that the HPV vaccine is not required for school (*p* < 0.001). In the first mediation analysis of the full sample (*n* = 137 at baseline, *n* = 120 at follow-up), the mediator variable significantly mediated an increase in UPBR use between baseline and follow-up (See Figure 1). In other words, non-use of UPBR at baseline was associated with significantly lower odds (B = −0.411, *p* < 0.000) of somewhat/strongly disagreeing with this belief (it is necessary to tell parents that the HPV vaccine is not required for school) after delivery of AVP. Pediatricians who strongly/somewhat disagreed with this belief at follow-up had significantly increased odds of also using UBPRs at follow-up (B = 0.392, *p* < 0.000). There was a significant non-mediating effect of the non-use of UPBRs at baseline on the use of UPBR at follow-up; non-use of UPBRs at baseline was associated with a lower likelihood of UPBR use at follow-up (B = −0.155, *p* = 0.001).

**Figure 2:**
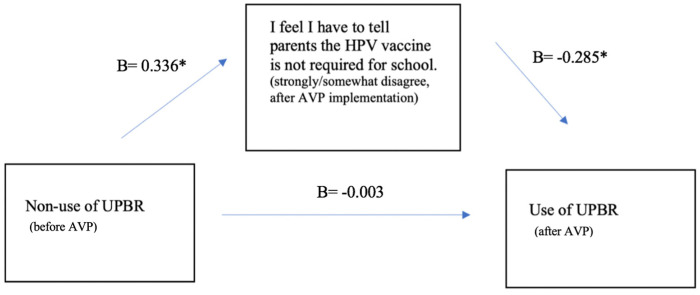
A mediation analysis of change in use of unqualified, bundled, presumptive recommendation (UPBR) before and after delivery of AVP, among subsample of matched pediatricians (*n* = 66). * denotes *p* < 0.05.

The second mediation analysis focused on the matched subsample of pediatricians who completed both the baseline and follow-up survey (see Figure 2). First, we observed a significant effect between non-use of UPRs at baseline (exogenous variable) and agreement with telling parents that HPV (mediation) was not required for school (B = 0.336, *p* = 0.003). Second, we found a significant effect between agreement with telling parents about HPV vaccine requirements (mediation) and non-use of UPRs at follow-up (B = −0.285, *p* = 0.002), controlling for non-use of UPRs at baseline (B = −0.003, *p* = 0.970). 

## 4. Discussion 

The results from this study indicate that, in addition to improving HPV vaccination rates [28], AVP implementation also significantly increased pediatrician delivery of UPBRs (unqualified, presumptive, bundled recommendations) within a large network of over 51 pediatric clinics and over 200 pediatricians. Pediatrician UPBR use is associated with increased uptake of the HPV vaccine among children ages 11–12 [11]. While public health communication guidelines call for pediatricians to use bundled, presumptive recommendations [13], few intervention studies have reported effective approaches to increasing providers’ delivery of strong HPV vaccination recommendations. AVP was effective in improving the quality of providers’ vaccine recommendations. AVP implementation also resulted in a statistically significant decrease in four pediatrician barriers and an increase in two facilitators to delivering UPBRs. The psychosocial barrier determinants that AVP reduced were: the belief that it is okay to delay HPV vaccination for 11–12-year-olds until they are older, concern about the financial burden of the HPV vaccine on parents, the belief that it is necessary to tell parents that the HPV vaccine is not required for school, and the belief that it is necessary to discuss issues of sexuality when recommending the HPV vaccine. The psychosocial facilitator determinants that AVP increased were: the belief that the severity of HPV-associated disease in males justifies the routine use of the HPV vaccine in males and the rated importance of ensuring that adolescents are up-to-date with HPV vaccination.

Our mediation models also highlight the extent to which one key belief plays a crucial role in influencing pediatricians’ likelihood of using UPBRs. Pediatricians’ shifts toward UPBR use between baseline and follow-up after the delivery of AVP were significantly mediated by pediatricians’ belief that it is necessary to tell parents that the HPV vaccine is not required for public school attendance. AVP implementation significantly reduced this pediatrician belief, and it was a statistically significant predictor of UPBR use. The mediation effect was statistically significant in both the entire sample and in the matched subsample of pediatricians (*n* = 66) who completed the baseline survey before AVP implementation and the follow-up survey after implementation. In other words, the change in this belief was the primary psychosocial mechanism of change responsible for increasing pediatricians’ use of UPBRs. This result aligns with previous qualitative research, finding that pediatricians may reference school requirements when qualifying their vaccine recommendation. Specifically, providers present the HPV vaccine as available, but, unlike other vaccines, highlight that it is not required for public school attendance [12,46]. Qualitative evidence has documented physicians’ frustration at school requirements; because letters sent to parents from schools do not mention the HPV vaccine, physicians fear that the discrepancy between their recommendations and the school’s recommendations creates confusion and distrust among parents [47]. Furthermore, political controversy surrounding legislative attempts to mandate HPV vaccination in schools and the persistent presence of political discourse about the HPV vaccine on social media [48,49,50,51] may also contribute to pediatricians’ school-related qualifications. Exposure to messages highlighting the politicization of the HPV vaccine is associated with lower trust in doctors [49]. It seems likely that doctors may qualify their HPV vaccine recommendations with acknowledgements of school policy in an effort to avoid being perceived as political and to garner trust with parents. Notably, AVP’s CME for pediatricians specifically address pediatrician beliefs about school requirements. Additionally, leadership communications about AVP emphasize the importance of basing recommendations on CDC guidelines, rather than school policies. Our results suggest that intervention strategies that address pediatricians’ concerns about the lack of school vaccination requirements, enacted in tandem with other system-level changes that support HPV vaccination, may be especially helpful in encouraging health care providers to deliver high-quality HPV vaccine recommendations. Both a recent meta-analysis and a systematic review of interventions designed to promote HPV vaccination did not include any reference to changing physicians’ feelings about disclosing school requirements as behavioral strategies used in interventions [52,53]. Future interventions aimed at increasing adolescent HPV vaccine uptake should devote specific focus to decreasing pediatricians’ beliefs about the necessity of qualifying the recommendation by discussing the non-mandatory nature of HPV vaccination for school attendance.

Additionally, our findings point to the potential benefit of policy-change efforts to add the HPV vaccine to public school vaccination requirements. The current policy for public school attendance in some US states does not require HPV vaccination, which is not in alignment with CDC recommendations that children ages 11–12 receive the HPV vaccine [54]. More research is needed to examine the importance of pediatricians’ beliefs about school policy more widely and to strengthen communication interventions focused on improving providers’ HPV vaccination recommendations.

Our study’s strengths include examining a wide range of psychosocial determinants of UPBRs at two time-points within a large sample of pediatricians within a 51-clinic network. Our study also has several limitations. Surveys were cross-sectional, meaning that baseline and follow-up samples were not matched. Of the 189 unique pediatricians who completed at least one survey, 36% completed both the baseline and follow-up surveys. However, we also conducted a mediation analysis with a matched subsample of pediatricians who completed both the baseline and follow-up surveys. The results of this analysis were also statistically significant, finding that the link between UPBR at baseline and UPBR at follow-up was completely mediated by pediatricians’ feelings about disclosing school vaccine requirements. The results of the analysis in the matched subsample confirmed the finding that pediatricians’ feelings about school requirements for vaccines are extremely important in shaping their willingness to use UPBRs to recommend the HPV vaccine.

AVP delivered a suite of evidence-based strategies to participating clinics. The current study analyzed the impact of AVP as a system-level intervention and did not distinguish between the effects of AVP’s different evidence-based strategies, including CME, vaccination reminders for pediatricians and parents, pediatrician assessment and feedback regarding vaccination rates, and designated “HPV immunization champions” for each clinic. Not all pediatricians who participated in the AVP study completed the CME, further indicating that AVP’s combination of evidence-based strategies, rather than education alone, drove pediatricians’ psychosocial changes. Of the pediatricians who responded to the baseline survey, 29.2% later completed the CME; 34.2% of physicians who responded to the follow-up survey completed the CME; and 32.4% of pediatricians who completed both baseline and follow-up surveys completed the CME. 

This study relies on a single-group pre–post-test design without a control group. Recall bias and social desirability response bias may have influenced pediatricians’ responses to survey questions. Implementation of AVP began in 2017 and ended in 2019, so some pediatricians may have completed the CME two years before receiving the follow-up survey. Additionally, the pediatricians surveyed were mainly white females (74.2% female, 48.3% white). It is possible that male pediatricians or pediatricians with different racial or ethnic backgrounds may vary in communication styles [55,56] and, therefore, experience different barriers to delivering UPBRs. Furthermore, because HPV vaccination policies, availability, and delivery mechanisms differ widely across countries [57], the results of this study may not be generalizable outside of the US. Two global systematic reviews of HPV vaccination strategies found no studies outside the US mentioning provider communication strategies [58,59]. Future research should continue to study barriers to effective communication about the HPV vaccine among more diverse samples of pediatricians in the US and in different countries.

## 5. Conclusions

Our findings have significant implications for future efforts to increase HPV vaccination among adolescents. AVP was successful in increasing the use of UPBRs among pediatricians. Change toward UPBR use is related to one critical psychosocial determinant: pediatricians’ beliefs about communication regarding the non-mandatory nature of HPV vaccination for school enrollment. This barrier contributes to missed vaccination opportunities and potentially reinforces parent hesitancy. This finding is consistent with behavioral theory and provides empirical evidence regarding mechanisms explaining AVP’s effectiveness in improving pediatricians’ communication about HPV vaccination. Self-efficacy to deliver recommendations and to persuade parents were not influential mediators toward UPBRs. Future interventions should address this essential pediatrician barrier to delivering presumptive HPV vaccination recommendations. 

## Figures and Tables

**Table 1:** Pediatrician characteristics at follow-up.

Characteristics	Mean	Range
Pediatrician age	47	30–87
Number of years at clinic	10	0–25
Year that residence training was completed	2002	1960–2018
**Pediatrician gender**	**N**	**%**
Male	31	25.8
Female	89	74.2
**Ethnicity**	**N**	**%**
Non-Hispanic White	58	48.3
Non-Hispanic Black	12	10
Hispanic	13	10.8
Asian	26	21.7
Other (including multiracial)	2	1.7
Prefer not to answer	9	7.5
**Number of patients seen on a typical day**	**N**	**%**
<15	0	0
15–19	4	3.3
20–29	65	54.2
30+	51	42.5

**Table 2:** Differences in psychosocial variables between baseline and follow-up.

Psychosocial Variable Bold Text Denotes *p* < 0.0015	Baseline Mean Rating (S.D.)	Follow-Up Mean Rating (S.D.)	t (DF)	*p*
*Pediatrician knowledge and attitudes* (α = 0.50)				
Your level of knowledge about HPV ^a^	1.25 (0.57)	1.15 (0.38)	1.65 (239.86)	0.101
Your concern about vaccine safety. ^a^	1.19 (0.48)	1.08 (0.28)	2.15 (223.09)	0.028
Your concern about vaccine efficacy. ^a^	1.11 (0.34)	1.06 (0.27)	1.36 (252.97)	0.177
**It is okay to delay HPV vaccination for 11–12-year-olds until they are older. ^b^**	**2.28 (0.84)**	**1.83 (0.81)**	**4.4 (254)**	**<0.00**
The severity of HPV-associated diseases in females justifies the routine use of the HPV vaccine in males. ^b^	3.66 (0.55)	3.61 (0.44)	0.68 (254)	0.495
**The severity of HPV-associated disease in males justifies the routine use of the HPV vaccine in males. ^b^**	**3.51 (0.62)**	**3.81 (0.44)**	**−4.42 (242.23)**	**<0.001**
At what age would you vaccinate your own child against HPV? ^c^	1.21 (0.55)	1.08 (0.34)	2.12 (239.34)	0.035
**To what extent do you feel it is important to ensure adolescents are up-to-date with HPV vaccination? ^d^**	**4.77 (0.47)**	**4.96 (0.20)**	**−4.22 (185.36)**	**<0.000**
*Pediatrician Perception of Parental Attitudes* (α = 0.703)				
Your concern about parents’ negative perceptions about the HPV vaccine. ^a^	2.26 (0.99)	2.07 (0.95)	1.56(255)	0.121
Parental concerns about vaccine efficacy. ^a^	2.02 (0.92)	2.08 (0.99)	−0.45 (255)	0.656
Parental concerns about vaccine safety. ^a^	3.12 (0.91)	3.23 (0.85)	−0.98 (255)	0.328
Parental mistrust of vaccines. ^a^	2.76 (0.88)	3.08 (0.86)	−2.99 (255)	0.003
Parental belief that child is not at risk for HPV infection. ^a^	3.21 (0.87)	3.13 (0.83)	0.82 (255)	0.415
Parental belief that child is too young for HPV vaccine. ^a^	3.37 (0.76)	3.1 (0.88)	2.66 (255)	0.008
Parents of 11–12-year-old patients are more likely to refuse the HPV vaccine than parents of 16–21-year-old patients. ^b^	3.22 (0.78)	2.97 (0.81)	2.56 (254)	0.011
*Pediatrician Concerns About Practicality* (α = 0.376)				
**Your concern about the financial burden of the HPV vaccine on patients. ^a^**	**1.28 (0.53)**	**1.07 (0.25)**	**4.31 (199.82)**	**<0.000**
The time it takes to discuss HPV vaccination with patients and parents. ^a^	1.93(0.86)	1.94 (0.87)	−0.14 (255)	0.892
Difficulty ensuring that patients will complete the 3-dose HPV vaccine series. ^a^	2.18 (0.95)	2.03 (0.95)	1.2 (255)	0.233
Infrequent office visits made by adolescent patients. ^a^	2.47 (0.95)	2.33 (0.97)	1.25 (255)	0.214
I feel I have enough time during appointments to probe parents about their reasons for wanting to refuse or delay HPV vaccination for their adolescent child. ^b^	2.43 (0.84)	2.56 (0.94)	−1.33 (254)	0.183
*Pediatrician Feelings About School Requirements* (α = −0.449)				
**I feel I have to tell parents the HPV vaccine is not required for school. ^b^**	**2.69 (1.04)**	**2.1 (1.02)**	**4.58 (254)**	**<0.000**
The HPV vaccine is not required for school attendance. ^a^	2.28 (1.05)	2.38 (1.09)	−0.8 (255)	0.428
*Pediatrician Perception of Physician Norms* (α = 0.297)				
I am expected to vaccinate children ages 11–12 against HPV according to the recommendations. ^b^	3.45 (0.76)	3.63 (0.77)	−1.85 (254)	0.066
The people in my profession whose opinions I value want me to vaccinate patients against HPV according to the recommendations. ^b^	3.66 (0.57)	3.63 (0.78)	0.34 (254)	0.737
*Pediatrician Communication Self-Efficacy* (α = 0.801)				
I am influential in parents’ final decision about whether to get the HPV vaccine for their child. ^b^	3.26 (0.58)	3.21 (0.65)	0.64 (254)	0.524
I am usually able to convince hesitant parents to get the HPV vaccine at the 11–12 year-old visit. ^b^	2.82 (0.76)	2.88 (0.76)	−0.71 (254)	0.482
I am confident I can address specific parental concerns about the HPV vaccine for girls. ^b^	3.72 (0.45)	3.6 (0.59)	1.79 (222.7)	0.07
I am confident I can address specific parental concerns about the HPV vaccine for boys.^b^	3.65 (0.54)	3.56 (0.58)	1.34 (253)	0.181
I am confident I can address parental concerns about HPV vaccine safety. ^b^	3.65 (0.54)	3.53 (0.65)	1.59 (232.4)	0.110
I am confident I can address parental concerns about vaccinating their child at age 11 or 12. ^b^	3.56 (0.57)	3.74 (0.48)	−2.73 (252.17)	0.007
I am confident I can explain the benefits of HPV vaccination to parents. ^b^	3.82 (0.40)	3.87 (0.41)	−0.87 (253)	0.383
*Pediatrician Self-Efficacy to Communicate About Sex* (α = −0.318)				
**It is necessary to discuss issues of sexuality when recommending the HPV vaccine to patients or parents of patients. ^b^**	**2.73 (0.88)**	**2.34 (0.97)**	**3.35 (254)**	**0.001**
I am confident I can address parental concerns that the HPV vaccine may increase adolescent sexual activity. ^b^	3.65 (0.60)	3.64 (0.63)	0.13 (253)	0.900
Your personal discomfort talking about sexually transmitted infections with parents and patients. ^a^	1.28 (0.53)	1.22 (0.43)	1.13 (253.98)	0.258

“a” superscript indicates the following survey response choices: 1 = Not at all a barrier, 2 = A minor barrier, 3 = Somewhat of a barrier, 4 = A major barrier; “b” superscript indicates the following survey response choices: 1 = Strongly disagree, 2 = Somewhat disagree, 3 = Somewhat agree, 4 = Strongly agree; “c” superscript indicates the following survey response choices: 1 = 11–12, 2 = 13–14, 3 = 15–18, 4 = I would not vaccinate my child against HPV. “d” superscript indicates the following survey response choices: 1 = Not very important, 2 = Somewhat unimportant, 3 = Neutral, 4 = Somewhat important, 5 = Very important.

**Table 3:** Psychosocial predictors of the use of the unqualified, presumptive, bundled recommendation.

	Odds Ratio	Standard Error	Z Statistic	*p*-Value	95% Confidence Interval	
					Lower	Upper
*Difficulty ensuring patients will complete the 3-dose HPV vaccine series*						
Not at all a barrier	Reference					
A minor barrier	0.915	0.388	−0.210	0.834	0.399	2.099
Somewhat of a barrier	0.648	0.291	−0.970	0.334	0.269	1.562
A major barrier	0.613	0.395	−0.760	0.447	0.174	2.164
*Parental concerns about vaccine efficacy a barrier to immunizing your patients against HPV*						
Not at all a barrier	Reference					
A minor barrier	0.866	0.339	−0.370	0.712	0.402	1.864
Somewhat of a barrier	0.779	0.369	−0.530	0.599	0.308	1.973
A major barrier	1.058	0.746	0.080	0.936	0.266	4.213
*Parental concerns about vaccine safety a barrier to immunizing your patients against HPV*						
Not at all a barrier	Reference					
A minor barrier	0.283	0.257	−1.390	0.164	0.048	1.675
Somewhat of a barrier	0.473	0.443	−0.800	0.425	0.075	2.970
A major barrier	0.426	0.404	−0.900	0.368	0.066	2.731
*Parental mistrust of vaccines a barrier to immunizing your patients against HPV*						
Not at all a barrier	Reference					
A minor barrier	1.097	0.877	0.120	0.908	0.229	5.256
Somewhat of a barrier	2.006	1.758	0.790	0.427	0.360	11.170
A major barrier	2.269	2.062	0.900	0.368	0.382	13.476
*It is necessary to discuss issues of sexuality when recommending the HPV vaccine to patients or parents.*						
Strongly disagree	Reference					
Somewhat disagree	1.511	0.779	0.800	0.423	0.550	4.149
Somewhat agree	1.110	0.538	0.220	0.829	0.429	2.871
Strongly agree	0.624	0.345	−0.850	0.393	0.211	1.843
*It is okay to delay HPV vaccination for 11–12-year-olds until they are older.*						
Strongly disagree	Reference					
Somewhat disagree	0.555	0.241	−1.360	0.174	0.237	1.299
*Somewhat agree*	0.282	0.123	−2.910	0.004	0.120	0.661
Strongly agree	0.608	0.625	−0.480	0.629	0.081	4.556
*I am expected to vaccinate children ages 11–12 against HPV according to the recommendations.*						
Strongly disagree	0.598	0.453	−0.680	0.498	0.136	2.644
Somewhat disagree	1.036	0.853	0.040	0.966	0.206	5.207
Somewhat agree	0.871	0.324	−0.370	0.711	0.420	1.806
Strongly agree	Reference					
*I feel I have to tell parents the HPV vaccine is not required for school.*						
Strongly disagree	Reference					
Somewhat disagree	0.385	0.211	−1.740	0.082	0.131	1.129
**Somewhat agree**	**0.117**	**0.063**	**−4.020**	**0.000**	**0.041**	**0.333**
**Strongly agree**	**0.107**	**0.059**	**−4.030**	**0.000**	**0.036**	**0.317**
Constant	31.087	30.999	3.450	0.001	4.403155	219.4744

The reference group was chosen as the level of each variable that represented the desirable outcome, i.e., expected to enhance the likelihood of successful HPV vaccination. Statistically significant results (*p* < 0.0015) are bolded for easier visualization. The variables listed in this table do not include the 26 variables that were removed due to multicollinearity.

## Data Availability

The data can be shared upon request.

## References

[1] Kreisel K.M., Spicknall I.H., Gargano J.W., Lewis F.M.T., Lewis R.M., Markowitz L.E., Roberts H., Johnson A.S., Song R., St Cyr S.B. (2021). Sexually Transmitted Infections Among US Women and Men: Prevalence and Incidence Estimates, 2018. Sex. Transm. Dis..

[2] Viens L.J. (2016). Human Papillomavirus–Associated Cancers—United States, 2008–2012. MMWR Morb. Mortal. Wkly. Rep..

[3] Meites E., Kempe A., Markowitz L.E. (2016). Use of a 2-Dose Schedule for Human Papillomavirus Vaccination—Updated Recommendations of the Advisory Committee on Immunization Practices. MMWR Morb. Mortal. Wkly. Rep..

[4] Centers for Disease Control and Prevention TeenVaxView. https://www.cdc.gov/teenvaxview/interactive/index.html.

[5] U.S. Department of Health and Human Services Healthy People 2030. https://health.gov/healthypeople/objectives-and-data/browse-objectives/vaccination.

[6] Pingali C., Yankey D., Elam-Evans L.D., Markowitz L.E., Valier M.R., Fredua B., Crowe S.J., Stokley S., Singleton J.A. (2022). National Vaccination Coverage Among Adolescents Aged 13–17 Years—National Immunization Survey-Teen, United States, 2021. MMWR Morb. Mortal. Wkly. Rep..

[7] Caldwell A.C., Madden C.A., Thompson D.M., Garbe M.C., Roberts J.R., Jacobson R.M., Darden P.M. (2021). The Impact of Provider Recommendation on Human Papillomavirus Vaccine and Other Adolescent Vaccines. Hum. Vaccin. Immunother..

[8] Gilkey M.B., Malo T.L., Shah P.D., Hall M.E., Brewer N.T. (2015). Quality of Physician Communication about Human Papillomavirus Vaccine: Findings from a National Survey. Cancer Epidemiol. Biomark. Prev..

[9] Oh N.L., Biddell C.B., Rhodes B.E., Brewer N.T. (2021). Provider Communication and HPV Vaccine Uptake: A Meta-Analysis and Systematic Review. Prev. Med..

[10] Willis D.E., Moore R., Selig J.P., Shafeek Amin N., Li J., Watson D., Brimberry R.K., McElfish P.A. (2024). Pediatric HPV Vaccination: Provider Recommendations Matter among Hesitant Parents. Vaccine.

[11] Savas L., Farias A., Healy C.M., Shegog R., Fernandez M.E., Frost E., Coan S., Crawford C., Spinner S., Wilber M. (2021). Wording Matters When Pediatricians Recommend HPV Vaccination. J. Appl. Res. Child..

[12] Shay L.A., Street R.L., Baldwin A.S., Marks E.G., Lee S.C., Higashi R.T., Skinner C.S., Fuller S., Persaud D., Tiro J.A. (2016). Characterizing Safety-Net Providers’ HPV Vaccine Recommendations to Undecided Parents: A Pilot Study. Patient Educ. Couns..

[13] Centers for Disease Control and Prevention HPV Vaccination: For Providers. https://www.cdc.gov/vaccines/vpd/hpv/hcp/index.html.

[14] Brewer N.T., Hall M.E., Malo T.L., Gilkey M.B., Quinn B., Lathren C. (2017). Announcements Versus Conversations to Improve HPV Vaccination Coverage: A Randomized Trial. Pediatrics.

[15] Bernstein T.A., Broome M., Millman J., Epstein J., Derouin A. (2022). Promoting Strategies to Increase HPV Vaccination in the Pediatric Primary Care Setting. J. Pediatr. Health Care.

[16] Brewer N.T., Mitchell C.G., Alton Dailey S., Hora L., Fisher-Borne M., Tichy K., McCoy T. (2021). HPV Vaccine Communication Training in Healthcare Systems: Evaluating a Train-the-Trainer Model. Vaccine.

[17] Fenton A.T., Eun T.J., Clark J.A., Perkins R.B. (2018). Indicated or Elective? The Association of Providers’ Words with HPV Vaccine Receipt. Hum. Vaccines Immunother..

[18] Opel D.J., Heritage J., Taylor J.A., Mangione-Smith R., Salas H.S., DeVere V., Zhou C., Robinson J.D. (2013). The Architecture of Provider-Parent Vaccine Discussions at Health Supervision Visits. Pediatrics.

[19] Byrne M., Curley C., Apple A., Clement E., Cory L. (2023). Every Nudge Counts: Impact of a Clinical Intervention Bundle on the Uptake of HPV Vaccination in an under-Resourced Clinic (051). Gynecol. Oncol..

[20] Farmar A.-L.M., Love-Osborne K., Chichester K., Breslin K., Bronkan K., Hambidge S.J. (2016). Achieving High Adolescent HPV Vaccination Coverage. Pediatrics.

[21] Keim-Malpass J., Mitchell E.M., Camacho F. (2015). HPV Vaccination Series Completion and Co-Vaccination: Pairing Vaccines May Matter for Adolescents. Vaccine.

[22] Zhu Y., Wu C.-F., Giuliano A.R., Fernandez M.E., Ortiz A.P., Cazaban C.G., Li R., Deshmukh A.A., Sonawane K. (2022). Tdap-HPV Vaccination Bundling in the USA: Trends, Predictors, and Implications for Vaccine Series Completion. Prev. Med..

[23] Kempe A., O’Leary S.T., Markowitz L.E., Crane L.A., Hurley L.P., Brtnikova M., Beaty B.L., Meites E., Stokley S., Lindley M.C. (2019). HPV Vaccine Delivery Practices by Primary Care Physicians. Pediatrics.

[24] Btoush R., Kohler R.K., Carmody D.P., Hudson S.V., Tsui J. (2022). Factors That Influence Healthcare Provider Recommendation of HPV Vaccination. Am. J. Health Promot..

[25] Farias A.J., Savas L.S., Fernandez M.E., Coan S.P., Shegog R., Healy C.M., Lipizzi E., Vernon S.W. (2017). Association of Physicians Perceived Barriers with Human Papillomavirus Vaccination Initiation. Prev. Med..

[26] Holman D.M., Benard V., Roland K.B., Watson M., Liddon N., Stokley S. (2014). Barriers to Human Papillomavirus Vaccination among US Adolescents: A Systematic Review of the Literature. JAMA Pediatr..

[27] Crawford C.A., Shegog R., Savas L.S., Frost E.L., Healy C.M., Coan S.P., Gabay E.K., Spinner S.W., Vernon S.W. (2019). Using Intervention Mapping to Develop an Efficacious Multicomponent Systems-Based Intervention to Increase Human Papillomavirus (HPV) Vaccination in a Large Urban Pediatric Clinic Network. J. Appl. Res. Child..

[28] Vernon S.W., Savas L.S., Shegog R., Healy C.M., Frost E.L., Coan S.P., Gabay E.K., Preston S.M., Crawford C.A., Spinner S.W. (2019). Increasing HPV Vaccination in a Network of Pediatric Clinics Using a Multi-Component Approach. J. Appl. Res. Child..

[29] Cataldi J.R., O’Leary S.T., Markowitz L.E., Allison M.A., Crane L.A., Hurley L.P., Brtnikova M., Beaty B.L., Gorman C., Meites E. (2021). Changes in Strength of Recommendation and Perceived Barriers to Human Papillomavirus Vaccination: Longitudinal Analysis of Primary Care Physicians, 2008–2018. J. Pediatr..

[30] Shefer A., Briss P., Rodewald L., Bernier R., Strikas R., Yusuf H., Ndiaye S., Wiliams S., Pappaioanou M., Hinman A.R. (1999). Improving Immunization Coverage Rates: An Evidence-Based Review of the Literature. Epidemiol. Rev..

[31] Vollrath K., Thul S., Holcombe J. (2018). Meaningful Methods for Increasing Human Papillomavirus Vaccination Rates: An Integrative Literature Review. J. Pediatr. Health Care.

[32] Eldredge L.K.B., Markham C.M., Ruiter R.A.C., Fernández M.E., Kok G., Parcel G.S. (2016). Planning Health Promotion Programs: An Intervention Mapping Approach.

[33] Healy C.M., Savas L.S., Shegog R., Lunstroth R., Vernon S.W. (2022). Medical Ethics Principles Underscore Advocating for Human Papillomavirus Vaccine. Hum. Vaccines Immunother..

[34] McRee A.-L., Gilkey M.B., Dempsey A.F. (2014). HPV Vaccine Hesitancy: Findings from a Statewide Survey of Health Care Providers. J. Pediatr. Health Care.

[35] Vadaparampil S.T., Kahn J.A., Salmon D., Lee J.-H., Quinn G.P., Roetzheim R., Bruder K., Malo T.L., Proveaux T., Zhao X. (2011). Missed Clinical Opportunities: Provider Recommendations for HPV Vaccination for 11–12 Year Old Girls Are Limited. Vaccine.

[36] McKenzie A., Savas L., Yeh P., Shegog R., Healy C.M., Shay L.A., Preston S., Frost E., Spinner S., Vernon S. Pediatrician psychosocial variables associated with HPV communication strategies. Proceedings of the Health Communication Division of the International Communication Association.

[37] Allison M.A., Dunne E.F., Markowitz L.E., O’Leary S.T., Crane L.A., Hurley L.P., Stokley S., Babbel C.I., Brtnikova M., Beaty B.L. (2013). HPV Vaccination of Boys in Primary Care Practices. Acad. Pediatr..

[38] Askelson N.M., Campo S., Lowe J.B., Dennis L.K., Smith S., Andsager J. (2010). Factors Related to Physicians’ Willingness to Vaccinate Girls against HPV: The Importance of Subjective Norms and Perceived Behavioral Control. Women Health.

[39] Bynum S.A., Staras S.A.S., Malo T.L., Giuliano A.R., Shenkman E., Vadaparampil S.T. (2014). Factors Associated with Medicaid Providers’ Recommendation of the HPV Vaccine to Low-Income Adolescent Girls. J. Adolesc. Health.

[40] Daley E.M., Perrin K.M., McDermott R.J., Vamos C.A., Rayko H.L., Packing-Ebuen J.L., Webb C., McFarlane M. (2010). The Psychosocial Burden of HPV: A Mixed-Method Study of Knowledge, Attitudes and Behaviors among HPV+ Women. J. Health Psychol..

[41] Feemster K.A., Winters S.E., Fiks A.G., Kinsman S., Kahn J.A. (2008). Pediatricians’ Intention to Recommend Human Papillomavirus (HPV) Vaccines to 11- to 12-Year-Old Girls Postlicensing. J. Adolesc. Health.

[42] McCave E.L. (2010). Influential Factors in HPV Vaccination Uptake among Providers in Four States. J. Community Health.

[43] Nelson D.E., Kreps G.L., Hesse B.W., Croyle R.T., Willis G., Arora N.K., Rimer B.K., Viswanath K.V., Weinstein N., Alden S. (2004). The Health Information National Trends Survey (HINTS): Development, Design, and Dissemination. J. Health Commun..

[44] Oster N.V., McPhillips-Tangum C.A., Averhoff F., Howell K. (2005). Barriers to Adolescent Immunization: A Survey of Family Physicians and Pediatricians. J. Am. Board Fam. Pract..

[45] Bland J.M., Altman D.G. (1995). Multiple Significance Tests: The Bonferroni Method. BMJ.

[46] Hughes C.C., Jones A.L., Feemster K.A., Fiks A.G. (2011). HPV Vaccine Decision Making in Pediatric Primary Care: A Semi-Structured Interview Study. BMC Pediatr..

[47] Tsui J., Vincent A., Anuforo B., Btoush R., Crabtree B.F. (2021). Understanding Primary Care Physician Perspectives on Recommending HPV Vaccination and Addressing Vaccine Hesitancy. Hum. Vaccines Immunother..

[48] McKenzie A.H., Avshman E., Shegog R., Savas L.S., Shay L.A. (2024). Facebook’s Shared Articles on HPV Vaccination: Analysis of Persuasive Strategies. BMC Public Health.

[49] Fowler E.F., Gollust S.E. (2015). The Content and Effect of Politicized Health Controversies. ANNALS Am. Acad. Political Soc. Sci..

[50] Saulsberry L., Fowler E.F., Nagler R.H., Gollust S.E. (2019). Perceptions of Politicization and HPV Vaccine Policy Support. Vaccine.

[51] Shay L.A., McKenzie A., Avshman E., Savas L.S., Shegog R. (2024). HPV Vaccine-Related Articles Shared on Facebook from 2019 to 2021: Did COVID Make a Difference?. PEC Innov..

[52] Escoffery C., Petagna C., Agnone C., Perez S., Saber L.B., Ryan G., Dhir M., Sekar S., Yeager K.A., Biddell C.B. (2023). A Systematic Review of Interventions to Promote HPV Vaccination Globally. BMC Public Health.

[53] Rodriguez A.M., Do T.Q.N., Goodman M., Schmeler K.M., Kaul S., Kuo Y.-F. (2019). Human Papillomavirus Vaccine Interventions in the U.S.: A Systematic Review and Meta-Analysis. Am. J. Prev. Med..

[54] Meites E., Szilagyi P.G., Chesson H.W., Unger E.R., Romero J.R., Markowitz L.E. (2019). Human Papillomavirus Vaccination for Adults: Updated Recommendations of the Advisory Committee on Immunization Practices. MMWR Morb. Mortal. Wkly. Rep..

[55] Roter D.L., Hall J.A., Aoki Y. (2002). Physician Gender Effects in Medical Communication: A Meta-Analytic Review. JAMA.

[56] Shen M.J., Peterson E.B., Costas-Muñiz R., Hernandez M.H., Jewell S.T., Matsoukas K., Bylund C.L. (2018). The Effects of Race and Racial Concordance on Patient-Physician Communication: A Systematic Review of the Literature. J. Racial Ethn. Health Disparities.

[57] Topazian H.M., Kundu D., Peebles K., Ramos S., Morgan K., Kim C.J., Richter K.L., Brewer N.T., Peris M., Smith J.S. (2018). HPV Vaccination Recommendation Practices among Adolescent Health Care Providers in 5 Countries. J. Pediatr. Adolesc. Gynecol..

[58] Efua Sackey M., Markey K., Grealish A. (2022). Healthcare Professional’s Promotional Strategies in Improving Human Papillomavirus (HPV) Vaccination Uptake in Adolescents: A Systematic Review. Vaccine.

[59] Singh P., Dhalaria P., Kashyap S., Soni G.K., Nandi P., Ghosh S., Mohapatra M.K., Rastogi A., Prakash D. (2022). Strategies to Overcome Vaccine Hesitancy: A Systematic Review. Syst. Rev..

